# An assessment of the knowledge, perception, and willingness to use telepharmacy services among the general public in Riyadh

**DOI:** 10.1097/MD.0000000000040853

**Published:** 2024-12-13

**Authors:** Mohammad Tarique Imam, Khaled AlAnazi, Amirah AlMakwini, Zyad Alahmadi, Mohamed Ali, Khaled AlQahtani, Saleh Alharbi, Nehad J. Ahmed

**Affiliations:** a Department of Clinical Pharmacy, College of Pharmacy, Prince Sattam bin Abdulaziz University, Al Kharj, Saudi Arabia; b College of Pharmacy, Prince Sattam bin Abdulaziz University, Al Kharj, Saudi Arabia.

**Keywords:** knowledge, perception, public, telepharmacy, use

## Abstract

The studies concerning Saudis’ perceptions and awareness of telepharmacy, as well as their readiness to use this service, are critical for policymakers to devise measures to increase the acceptability of telepharmacy. The present study aimed to assess their knowledge, perceptions, and willingness to use telepharmacy services in Riyadh. The present study was a cross-sectional survey conducted between March 2024 and June 2024. The data were obtained from the public via an online questionnaire built with Google Forms. The survey was filled out by 405 participants. Most of them were males (66.17%), and the ages of 56.78% of them were between 18 and 25 years. More than half of the participants had a bachelor’s degree (66.17%). More than half of the respondents said that they had heard about telepharmacy (61.73%), but 80.74% of them hadn’t used telepharmacy services before. Most of the respondents (78.52%) said that they like using telepharmacy services wherever possible, and 81.98% of them agreed that telepharmacy improves medication adherence. The findings of this study demonstrated a fair knowledge, positive perception, and high willingness to use telepharmacy among the general population in Riyadh. Interventions to increase knowledge of telepharmacy in Riyadh need to target less educated people.

## 1. Introduction

Telehealth is the remote delivery of medical and healthcare services utilizing information technology and communication tools.^[1]^ Telehealth encompasses a variety of topics, including telepharmacy, which refers to the use of telecommunications to provide patients with pharmacological treatment.^[2]^ This virtual approach to healthcare provision can be especially beneficial for patients living in remote locations where proper pharmaceutical care services are not accessible or cannot be offered in person.^[3]^

Telepharmacy is described as “a method used in pharmacy practice in which a pharmacist utilizes telecommunications technology to oversee aspects of pharmacy operations or provide patient-care services.”^[4]^ Telepharmacy shows the ability to enhance pharmacy services by lowering the number of medication-related issues, such as medication errors and adverse drug events.^[5]^ Furthermore, telepharmacy may have significant benefits for those living in rural regions or in locations with limited medical facilities and expert services.^[6]^ Telepharmacy is particularly useful when a pharmacy expert is required but not accessible in person.^[6]^

Telepharmacy benefits patients by reducing treatment-related difficulties, increasing medication adherence, reducing adverse drug events, and improving medication usage outcomes, all of which improve the quality of pharmacy services.^[7,8]^ It minimizes hospital admissions, reduces hospital burden, and saves time for patients and staff.^[9–11]^ Telepharmacy enables a tailored connection between the patient and the pharmacist, which includes patient-care services such as counseling, medication adherence evaluation, and thorough medication review.^[2,7,12]^

Many community pharmacists in Jordan consider that telepharmacy enables patients to get prompt medical advice.^[13]^ Previous studies in the United States and Jordan found that pharmacy students are unaware of telepharmacy but agree that it would be advantageous for avoiding medication-related difficulties and saving time, yet they express a strong willingness to use the services.^[13,14]^ Regulations, patients’ access to technology, and financial position may influence people’s decision to use telepharmacy services.^[15]^ Nonetheless, there is a lack of studies concerning Saudis’ perceptions and awareness of telepharmacy, as well as their readiness to use this service. This research is critical for policymakers devising measures to increase the acceptability of telepharmacy. As a result, we sought to assess the general public’s knowledge, perceptions, and willingness to use telepharmacy services in Riyadh region in the Kingdom of Saudi Arabia.

## 2. Materials and methods

We conducted a cross-sectional survey of the Saudi general population between March 2024 and June 2024. Anonymous data were obtained from the public via an online questionnaire built with Google Forms, which included questions on age, gender, level of education, knowledge, willingness, and perceptions about telepharmacy.

The sample size was determined using Slovin’s method, and a minimum of 156 participants was necessary to achieve a 95% confidence level and an 8% margin of error based on Saudi Arabia’s total population of 34 million.^[16–18]^

Slovin’s formula is calculated as: n = N/(1 + N*e*2),

where n: sample size needed, N: population size, *e*: acceptable margin of error.

A 13-item self-administered questionnaire was provided to people residing in Saudi Arabia. This questionnaire was derived from prior studies.^[19,20]^ The knowledge and willingness questions were answered with either Yes or No. Participants’ perceptions were examined using a 5-point Likert scale ranging from 1 (strongly disagree) to 5 (strongly agree). Social media platforms such as Twitter, Facebook, and WhatsApp were used to contact the public.

We examined the data descriptively and provided the results as numbers and percentages. We conducted *t* tests and logistic regression tests to determine the factors associated with the public’s knowledge and perception about telepharmacy. Statistical software Jamovi was used for analysis.

## 3. Results

The survey was filled out by 405 participants. Most of them were males (66.17%) and the ages of 56.78% of them were between 18 and 25 years. More than half of the participants had a bachelor’s degree (66.17%; Table 1).

**Table 1:** Demographic characteristics of the participants.

Variable	Category	Number	Percentage
Gender	Male	268	66.17
	Female	137	33.83
Level of education	Middle school	18	4.44
	High school	77	19.01
	Diploma	25	6.17
	Undergraduate	268	66.17
	Master’s degree	11	2.72
	Doctoral degree	6	1.48
Age	18–25	230	56.79
	26–35	109	26.91
	36–45	44	10.86
	46–55	16	3.95
	>55	6	1.48

More than half of the respondents said that they had heard about telepharmacy (61.73%) but 80.74% of them didn’t use telepharmacy services before. Only 37.78% of the respondents agreed that telepharmacy does require a strong internet connection or high-performance technology. Most of the respondents are interested in using telepharmacy services (88.88%; Table 2).

**Table 2:** Knowledge and willingness to use telepharmacy services.

Item	Category	Number	Percentage
Have you ever heard about telepharmacy?	Yes	250	61.73
	No	155	38.27
Have you ever used telepharmacy services before?	Yes	78	19.26
	No	327	80.74
Telepharmacy does require a strong internet connection or high-performance technology.	Yes	153	37.78
	No	252	62.22
Patients from rural areas can have more medication access and information via telepharmacy.	Yes	267	65.93
	No	138	34.07
Are you interested in using telepharmacy services?	Yes	360	88.88
	No	45	11.11

Most of the respondents (78.52%) said that they like using telepharmacy services and 91.35% of them agreed that telepharmacy service is important to be able to communicate with medical practitioners whenever and wherever. Most of the respondents agreed that telepharmacy helps to save time and energy (89.63%), improves medication adherence (81.98%), and reduces service costs (85.68%; Table 3).

**Table 3:** Perception of telepharmacy services.

Item	Category	Number	Percentage
There are many telepharmacy services that I can use in Saudi Arabia	Strongly agree	176	43.46
	Agree	102	25.18
	Neutral	107	26.42
	Disagree	11	2.72
	Strongly disagree	9	2.22
I like using telepharmacy services	Strongly agree	225	55.56
	Agree	93	22.96
	Neutral	62	15.31
	Disagree	23	5.68
	Strongly disagree	2	0.49
Telepharmacy service is important to be able to communicate with medical practitioners whenever and wherever	Strongly agree	284	70.12
	Agree	86	21.23
	Neutral	33	8.15
	Disagree	1	0.25
	Strongly disagree	1	0.25
Telepharmacy helps to save time and energy	Strongly agree	287	70.86
	Agree	76	18.77
	Neutral	21	5.19
	Disagree	20	4.94
	Strongly disagree	1	0.24
Telepharmacy improves my adherence to the medication	Strongly agree	188	46.42
	Agree	144	35.56
	Neutral	48	11.85
	Disagree	22	5.43
	Strongly disagree	3	0.74
Telepharmacy helps to reduce service costs	Strongly agree	267	65.93
	Agree	80	19.75
	Neutral	36	8.89
	Disagree	6	1.48
	Strongly disagree	16	3.95
I am willing to pay for telepharmacy services	Strongly agree	151	37.28
	Agree	61	15.06
	Neutral	109	26.91
	Disagree	41	10.12
	Strongly disagree	43	10.62
I will recommend telepharmacy to my friends and family	Strongly agree	265	65.43
	Agree	99	24.44
	Neutral	37	9.14
	Disagree	4	0.99
	Strongly disagree	0	0

Table 4 shows the knowledge and perception scores of the respondents. More than 51% of the respondents had poor knowledge and only 30.62% of them had good knowledge. The average score of knowledge was 54.72% (fair knowledge). Most of the respondents (88.89%) had a positive perception. The average score of perception was 79.75% (positive perception).

**Table 4:** Knowledge and perception scores of the respondents.

The score of knowledge*	N (%)
Poor knowledge	208 (51.36%)
Fair knowledge	73 (18.02%)
Good knowledge	124 (30.62%)

The score of perception†	N (%)
Positive perception	360 (88.89%)
Negative perception	45 (11.11%)

* Poor knowledge (score < 50%), fair knowledge (score 50–75%), good knowledge (score > 75).

† Positive perception (score > 50%), negative perception (score < 50%).

The results of the logistic regression analysis in Table 5 show that the public’s knowledge of telepharmacy was significantly associated with age. Public knowledge of telepharmacy was found to be correlated with education level, however, this correlation was not statistically significant (*P* value was .341).

**Table 5:** Factors associated with the public’s knowledge about telepharmacy.

Item	Category	Total	Average score (%)	*P* value* (*t* test)	*P* value** (logistic regression)
Gender	Males	268	53.8	.295	.295
	Female	137	56.5		
Education level	Secondary school	95	56.2	.341	.341
	Diploma	25	57.6		
	Bachelor	268	53.5		
	Postgraduate	17	60.0		
Age	18–25	230	46.4	**<.001**	**<.001**
	26–35	109	66.4		
	36–45	44	62.7		
	>45	22	67.0		

* *P* value of *t* test.

** *P* value of logistic regression.

The results of the logistic regression analysis in Table 6 show that the public’s perception of telepharmacy was strongly associated with education level and the respondents’ age.

**Table 6:** Factors associated with the public’s perception of telepharmacy.

Item	Category	Total	Average score (%)	*P* value* (*t* test)	*P* value** (logistic regression)
Gender	Males	268	80.5	.375	.375
	Female	137	78.2		
Education level	Secondary school	95	70.1	**<.001**	**<.001**
	Diploma	25	80.5		
	Bachelor	268	83.3		
	Postgraduate	17	75.0		
Age	18–25	230	77.5	**.003**	**.004**
	26–35	109	79.5		
	36–45	44	88.9		
	>45	22	85.2		

* *P* value of *t* test.

** *P* value of logistic regression.

## 4. Discussion

Telepharmacy services are very valuable because they improve access to healthcare, help manage medications, reduce costs, and support public health. By using technology, telepharmacy can make healthcare more efficient and focused on patients. It’s important to understand how well the public knows about telepharmacy, their opinions on it, and their willingness to use it. This knowledge can help improve healthcare delivery, encourage people to stick to their medication plans, enhance patient outcomes, and ensure telepharmacy meets the community’s needs. Such insights can guide policy decisions and improve the overall healthcare system.

About half of the respondents (50.5%) were aware of telepharmacy, but 75% had never used its services. A study by Jiptoatmadja and Alfian in Indonesia found that 51% had heard of telepharmacy, and most who hadn’t used it were interested in trying it in the future.^[19]^ This interest is higher than a similar study in India, where only 18.9% showed interest.^[21]^ In Jordan, a study by Abu-Farha et al^[22]^ found that 42.9% of participants had never heard of telepharmacy, and only 16.4% had used it.The difference between awareness and usage highlights a significant gap, showing the need for targeted promotion of telepharmacy. We can increase usage through educational campaigns, removing barriers, providing incentives, and encouraging collaboration.^[23]^

Most respondents believed that telepharmacy services are essential for communicating with healthcare providers. They also agreed that telepharmacy saves time and energy, improves medication adherence, and reduces costs. A study by Abu-Farha et al^[22]^ found that 83.5% of Jordanians said that the main benefit of telepharmacy is reducing unnecessary visits to pharmacies, while 79.0% valued the time and money saved on travel. Additionally, telepharmacy offers safety during pandemics and convenience for patients in remote areas.^[22]^ These positive views align with other studies on telepharmacy and telemedicine.^[24–29]^ Overall, the supportive feedback highlights the several advantages of telepharmacy, suggesting it can significantly enhance healthcare by improving communication, saving time, improving medication adherence, and reducing costs. Multiple studies support the potential of telepharmacy to change the healthcare sector.

The present study found also that people’s knowledge of telepharmacy is strongly associated with their age, while their perception of telepharmacy is associated with both age and education level. Tjiptoatmadja and Alfian noted a significant association between age and education and understanding of telepharmacy in Indonesia.^[19]^ However, they found no associations between demographic factors and attitudes toward using telepharmacy. Similarly, Alnajrani et al^[30]^ reported that in Saudi Arabia, knowledge of telepharmacy is associated with gender and education, but perceptions were not affected by age, gender, or education. Therefore, awareness campaigns in Riyadh should target older individuals and those with lower education to improve perceptions of telepharmacy.

To our knowledge, this is the first comprehensive evaluation of people’s knowledge, perceptions, and readiness to utilize telepharmacy and the factors associated with it in Riyadh. However, the study had limitations. The first drawback was that the study results were limited in generalizability due to the convenience sample approach. Convenience sampling selects individuals depending on their availability and desire to participate. This can generate bias since the sample is not always representative of the total population. Additionally, it is not random. This might result in a sample that does not accurately represent the population. Furthermore, data collected via online questionnaires have numerous methodological limitations. For example, some groups are less likely to have internet access and reply to online questionnaires. Additionally, the lack of a trained interviewer to clarify the information presented might result in less reliable results.

## 5. Conclusion

The findings of this study demonstrate a fair knowledge, positive perception, and high willingness to use telepharmacy among the general population in Riyadh. To increase knowledge of telepharmacy in Riyadh, interventions must target less educated people. Implementing interventions that increase the public’s knowledge and perception of telepharmacy in Riyadh is recommended. These interventions need to target older adults and people who are less educated.

## Acknowledgments

The authors extend their appreciation to Prince Sattam bin Abdulaziz University for funding this research work through the project number (PSAU/2024/03/29183).

## Author contributions

**Conceptualization:** Mohammad Tarique Imam, Nehad J. Ahmed.

**Data curation:** Khaled AlAnazi, Zyad Alahmadi, Khaled AlQahtani.

**Formal analysis:** Nehad J. Ahmed.

**Methodology:** Mohammad Tarique Imam.

**Software:** Amirah AlMakwini, Mohamed Ali, Saleh Alharbi.

**Supervision:** Nehad J. Ahmed.

**Writing – original draft:** Nehad J. Ahmed.

**Writing – review & editing:** Mohammad Tarique Imam.
